# Clinical Features and Outcomes of Neuropsychiatric Systemic Lupus Erythematosus in China

**DOI:** 10.1155/2021/1349042

**Published:** 2021-01-18

**Authors:** Shangzhu Zhang, Meng Li, Li Zhang, Ziqian Wang, Qiang Wang, Hui You, Yanhong Wang, Mengtao Li, Xiaofeng Zeng

**Affiliations:** ^1^Department of Rheumatology, Peking Union Medical College Hospital, Peking Union Medical College & Chinese Academy of Medical Sciences, National Clinical Research Center for Dermatologic and Immunologic Diseases, Ministry of Science & Technology, Key Laboratory of Rheumatology and Clinical Immunology, Ministry of Education, No. 1 Shuaifuyuan, Beijing 100730, China; ^2^Department of Radiology, Peking Union Medical College Hospital, Peking Union Medical College & Chinese Academy of Medical Sciences, No. 1 Shuaifuyuan, Beijing 100730, China; ^3^Department of Epidemiology and Biostatistics (YW), Institute of Basic Medical Sciences, Peking Union Medical College & China Academy of Medical Sciences, Beijing 100730, China

## Abstract

**Objective:**

To identify the clinical characteristics, magnetic resonance imaging (MRI) results, and prognostic factors of neuropsychiatric (NP) systemic lupus erythematosus (SLE; NPSLE) in a relatively large patient series in China.

**Methods:**

Data of patients with NPSLE at Peking Union Medical College Hospital (PUMCH) were collected retrospectively from June 2012 to June 2016. NPSLE patients were compared with 220 non-NPSLE patients. Survival rates were evaluated using the Kaplan-Meier curves, log-rank test, and Cox proportional hazards modeling. Cranial MRI results were also studied.

**Results:**

Of the 194 included patients, sixteen subtypes of NPSLE were identified, and the most common manifestations were seizure (36.6%), acute confusional state (25.3%), and cerebral vascular disease (15.5%). Compared with the non-NPSLE group, NPSLE patients were significantly more likely to have typical lupus symptoms, higher Systemic Lupus Erythematosus Disease Activity Index 2000 (SLEDAI-2K) scores (*P* = 0.002), and positive rate of anti-ribosomal P protein antibodies (*P* = 0.008). Patients with seizure were more likely to have higher SLEDAI-2K scores and positive anti-*β*2GP1 than non-NPSLE patients. Sixteen patients died during follow-up. The most common cause of death was infection (37.5%). NPSLE significantly decreased survival rates of SLE patients. Patients with elevated serum creatinine (*P* = 0.001), hypocomplementemia (*P* = 0.031), and SLEDAI − 2K scores ≥ 15 (*P* = 0.014) had shorter survival periods. Eighty-two patients underwent detailed cranial MRI analysis; of these, 50 (61.0%) had abnormal results. Small vessel disease was the most common abnormal finding, followed by inflammatory-like lesions and large vessel disease.

**Conclusions:**

High disease activity and positive rate of anti-ribosomal P protein antibodies may be risk factors for NPSLE. NPSLE decreases survival rates of SLE patients. Renal insufficiency and high disease activity are predictive of poor prognoses for NPSLE patients.

## 1. Introduction

Systemic lupus erythematosus (SLE) is an autoimmune disease involving multiple organ systems. Neuropsychiatric (NP) involvement is one of the most serious disorders in SLE and is usually associated with a poor prognosis [1]. The incidence of neuropsychiatric systemic lupus erythematosus (NPSLE) ranges from 12.2% to 94.7% for SLE patients [2, 3]. This wide range is probably due to the high variability of NP presentations and differences in study designs. The diversity and heterogeneity of NP presentations suggest that multiple pathogenetic mechanisms are involved in NPSLE. Previous studies showed that high disease activity is likely associated with diffuse central nervous system (CNS) NP manifestations [4, 5], and antiphospholipid antibody positivity is believed to be associated with cerebrovascular events [6]. Currently, research efforts are focusing on the identification of pathways involved in NPSLE development, including antibodies, cell-related inflammation, cytokine-related inflammation, and complement activity [7]. Seizure is one of the common subtypes of NPSLE. The underlying pathogenesis of seizure may be multifactorial and may include infarction, inflammatory mediators, and autoantibodies [8]. A previous study suggested that seizure is predictive of poor prognoses for SLE. Cranial magnetic resonance imaging (MRI) is still the most commonly used imaging technique for the detection and evaluation of NPSLE in clinical practice [9]. Whether abnormal or specific cranial MRI results can indicate the prognosis for NPSLE is questionable. There are many areas of NPSLE that have not yet been clarified. Furthermore, NPSLE leads to a decline in the quality of life and can be life-threatening. Therefore, NPSLE requires further study.

Because there have been few extensive studies of NPSLE in China, this study comprehensively analyzed the risk factors and short- to midterm outcomes of NPSLE in a large dataset of NPSLE patients in China. Clinical features of seizure and the relationship between cranial MRI and the prognosis for NPSLE were also evaluated.

## 2. Methods

### 2.1. Patient Recruitment

NPSLE inpatients and outpatients treated at Peking Union Medical College Hospital between June 2012 and June 2016 were considered. SLE was diagnosed when the SLE classification criteria recommended by the American College of Rheumatology (ACR) in 1997 [10] or those of the Systemic Lupus International Collaborating Clinics (SLICC) in 2012 [2] and the diagnostic criteria for NPSLE in SLE proposed by the ACR in 1999 were fulfilled [11]. The ACR defined 19 neurological syndromes (12 central and 7 peripheral NP), of which diffuse CNS disease included anxiety disorder, psychosis, mood disorder, cognitive dysfunction, and acute confusional states [11]. In the ACR case definitions [11], headache is defined using the International Headache Society (IHS) classification [12], and mood disorders are determined by clinical judgment based on the Diagnostic and Statistical Manual-IV (DSM-IV) criteria [13]. NP events caused by primary central nervous system diseases, central nervous system infections, metabolic abnormalities, electrolyte imbalance, trauma, degenerative disease, neoplasm, toxic exposure, major substance abuse, and primary psychiatric diseases were excluded. NP events were new or worsened at baseline. The control group was comprised of 220 SLE patients without NPSLE treated at Peking Union Medical College Hospital at the same time. The control group was matched for sex, age, and disease duration.

### 2.2. Data Collection

Clinical data were retrospectively collected, including demographic data (sex, age at enrollment, SLE duration, and NP duration), systemic manifestations, physical signs (including neurological signs), and laboratory examination results, including complete blood count, blood biochemical examination, immunoglobulin, complement, and anti-nuclear antibody (ANA) spectrum, including anti-ribosomal P protein antibody and antiphospholipid (aPL) antibody results consisting of anticardiolipin (aCL), anti-*β*2 glycoprotein 1, and lupus anticoagulant (LA). Dilute Russell viper venom time (dRVVT) was used to test LA. Cerebrospinal fluid (CSF) was collected and biochemical analyses and cranial MRI were performed. Data regarding the treatment and prognosis were also compiled. All the above manifestations and laboratory test results were recorded at the time of the NP event for NPSLE patients and at enrollment for the non-NPSLE patients. The SLE disease activity index (SLEDAI) was used to evaluate lupus activity [14]. SLEDAI ≥ 15 points indicates severe SLE activity [14]. The follow-up period was identified as the time of enrollment until the time of death or last follow-up examination. The survival time of NPSLE patients was identified as the period from the new onset of NP manifestations to the end of follow-up. The worsened patients were excluded from this analysis. Causes of death were determined by reviewing the case records and discussions with the attending physicians [15].

### 2.3. Cranial MRI Data Collection

The MRI results of all patients included T1-weighted imaging (T1WI), T2-weighted imaging (T2WI), fluid-attenuated inversion recovery (FLAIR) pulse sequence, diffusion-weighted imaging (DWI), and apparent diffusion coefficient (ADC). Some patients underwent perfusion-weighted imaging and enhanced T1WI. All MRI results were read and reported by two neuroradiologists who did not know the clinical manifestations, examination results, and prognosis information of the patients. Abnormal cranial MRI results were divided into inflammatory lesions, large vessel disease (LVD), and small vessel disease (SVD). Inflammatory lesions, defined as high signal on T1WI/FLAIR, can involve gray matter or white matter, are medium in size, have unclear boundaries and nonvascular distribution, and may have an occupying effect. LVD, defined as cerebral infarction in the great artery area, was described according to the number of lesions (single/multiple), related artery, and status (acute/chronic). According to the standards for reporting vascular changes observed on neuroimaging (stream), SVD was divided into white matter hyperintensity (WMH; including the basal ganglia and subtentorial area), short-term subcutaneous small infarcts, luminal infarction, microbleeding, and brain atrophy [16].

### 2.4. Statistical Analysis

SPSS 23.0 software and GraphPad Prism 8.0 were used for statistical analyses. Continuous data are expressed as mean ± standard deviation (SD). An independent sample *t*-test was used to compare variables between the two groups. For non-normally distributed data, the Mann–Whitney *U* test was used. Categorical data are expressed as numbers or percentages. The chi-square test or the Fisher's exact test was used to analyze the relationship between categorical variables. *P* < 0.05 indicated statistical significance. A multivariate logistic stepwise regression was performed with variables with a *P* value less than 0.05 in univariate analysis. The Kaplan-Meier method was used for survival analyses, and the log-rank test was used to compare survival rates. The Cox proportional hazard model was adopted to analyze predicting factors for mortality.

## 3. Results

### 3.1. Clinical Characteristics of NPSLE Patients and NPSLE Subtypes

Data from a total of 194 NPSLE patients were collected; 180 (92.8%) patients were female, and the average age was 29.9 ± 10.8 years. The median duration of SLE was 16 months (range, 0-361 months). The median duration of NP was 0.5 months (range, 0-191 months). NP involvement was the first symptom of 92 (47.4%) patients. Of all NPSLE patients, 184 (94.8%) had central nervous system (CNS) involvement, 21 (10.8%) had peripheral nervous system (PNS) involvement, and 11 (5.7%) had both CNS and PNS involvement. There were 16 subtypes of NPSLE. Seventy-one patients (36.6%) had more than one NP subtype. The most common subtypes are seizure, acute confusional state (ACS), cerebrovascular disease, headache, and psychosis (Table 1). Regarding laboratory examination results, 153 patients (78.9%) had hypocomplementemia during the course of the disease, 192 (99%) patients had positive antinuclear antibody (ANA), 97 patients (50.0%) had positive anti-dsDNA antibody, 81 patients (41.8%) had positive antiribosomal P protein antibody, and 58 patients (29.9%) had positive antiphospholipid antibody (aPL). The average SLEDAI score of NPSLE patients was 20.3 ± 9.1 points (range, 0-44 points); after removing the nervous system score, the average SLEDAI score was 12.3 ± 6.6 points (range 0-28 points). Lumbar puncture and CSF examinations were completed for all patients; 73 patients (37.6%) had elevated CSF pressure, and 78 patients (40.2%) had elevated CSF protein levels. Steroid pulse therapy (intravenous drip of methylprednisolone 1000 mg daily for three consecutive days) was administered to 146 patients (75.3%), which continued with oral prednisone (1 mg/kg·d) for 4 weeks; then, the steroid was tapered down. Immunosuppressive therapy was administered to 191 patients (98.5%); of these, 179 (92.3%) received cyclophosphamide (CTX) alone, 9 (4.6%) received mycophenolate mofetil (MMF) alone (Table 1), and 6 (3.1%) were administered combined immunosuppressive agents. Furthermore, 18 (9.3%) patients were on anticoagulation or antiplatelet treatment, 13 (6.7%) patients were on antiepileptic drugs, and 26 (13.4%) patients were on antipsychotics.

### 3.2. Comparison of Clinical Characteristics of the NPSLE Group and Non-NPSLE Group

Compared to the control group, NPSLE patients had a significantly higher likelihood of also having malar rash (*P* < 0.001), oral ulcer (*P* < 0.001), alopecia (*P* = 0.001), arthritis (*P* = 0.004), serositis (*P* < 0.001), renal disorder (*P* = 0.001), and fever (*P* < 0.001) than non-NPSLE patients. Leukopenia (*P* < 0.001), thrombocytopenia (*P* < 0.001), and hypocomplementemia (*P* < 0.001) significantly more frequently occurred in NPSLE patients than in the control group. The SLEDAI-2K score after removing the NP scores of the NPSLE group was significantly higher than that of non-NPSLE group (12.3 ± 6.8 vs. 8.7 ± 6.7, *P* < 0.001). Furthermore, the erythrocyte sedimentation rate (ESR) (*P* < 0.001) and CRP (*P* < 0.001) levels of the NPSLE group were significantly higher than those of the non-NPSLE group. The positive rate of anti-ribosomal P protein antibodies in the NPSLE group was significantly higher than that in the non-NPSLE group (41.8% vs. 21.4%, *P* < 0.001), but the positive rates of the anti-Sm antibodies and anti-SSB antibodies in the NPSLE group were significantly lower (35.6% vs. 51.8%, *P* = 0.001 and 17.5% vs. 32.7%, *P* = 0.001, respectively) than those of the non-NPSLE group. There was no difference in the positive aPL rates of the two groups (Table 2). In the logistic regression analysis, compared with the non-NPSLE group, the NPSLE group had more frequent malar rash (OR = 3.30, 95% CI 1.91–5.68, *P* < 0.001), serositis (OR = 3.98, 95% CI 2.12–7.45, *P* < 0.001), thrombocytopenia (OR = 2.10, 95% CI 1.17–3.78, *P* = 0.013), SLEDAI scores (excluding NP manifestations) ≥ 15 (OR = 2.97, 95% CI 1.50–5.90, *P* = 0.002), and positive rate of anti-ribosomal P protein antibodies (OR = 2.01, 95% CI 1.20–3.37, *P* = 0.008).

### 3.3. Prognostic Analysis of Clinical Characteristics of NPSLE Patients

**Figure 1:**
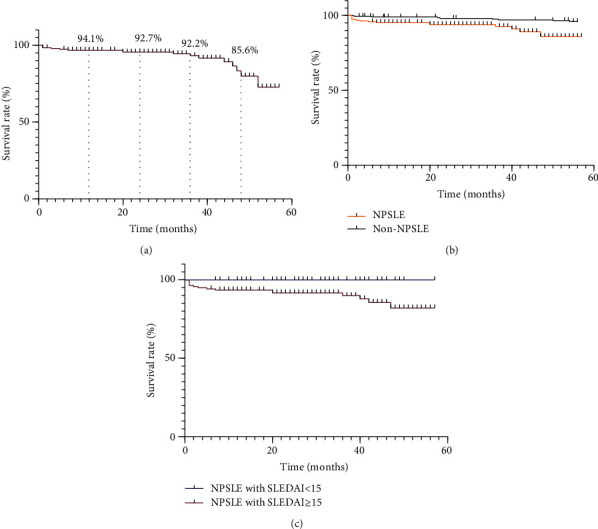
(a) The overall 1-, 2-, 3-, and 4-year survival rates of NPSLE patients. (b) Survival rates of NPSLE patients and non-NPSLE patients. The survival rate of NPSLE patients is significantly lower than that of non-NPSLE patients (*P* = 0.001). (c) Survival rates of NPSLE patients with SLEDAI score < 15 and SLEDAI score ≥ 15. The survival rate of NPSLE patients with SLEDAI score < 15 was significantly higher than that of patients with SLEDAI score ≥ 15 (*P* = 0.014).

The average survival time of NPSLE patients was 35.5 ± 23.5 months. Among the 194 patients, 16 died (1 male and 15 female patients). The overall 1-, 2-, 3-, and 4-year survival rates of NPSLE patients were 94.1% (95% confidence interval (CI), 92.5%-95.7%), 93.0% (95% CI, 91.3%-94.7%), 92.2% (95% CI, 90.0%-94.4%), and 85.6% (95% CI 81.2%-90.0%), respectively (Figure 1). The main causes of death were infection (6 patients; 37.5%), SLE-related causes (5 patients; 31.25%: pulmonary hypertension for 2 patients; nervous system involvement for 2 patients, and sudden cardiac death for 1 patient), and unknown cause (5 patients; 31.25%). The average follow-up time from enrolment to the endpoint for non-NP SLE patients was 50.2 ± 11.7 months. Eight patients died in the non-NPSLE group. The survival rate of the NPSLE group was significantly lower than that of the non-NPSLE group (*P* = 0.001) (Figure 1). NPSLE patients with a SLEDAI score ≥ 15 (*P* = 0.014), proteinuria (*P* = 0.035), elevated serum creatinine (Scr) (*P* = 0.001), and hypocomplementemia (*P* = 0.031) had shorter survival periods (Table 3; Figure 1). On multivariate analysis, elevated Scr (*P* = 0.029) was an independent prognostic factor of death (Table 4).

### 3.4. Comparison of Clinical Characteristics and Prognosis for NPSLE Patients with Seizure and Non-NPSLE Patients and NPSLE Patients without Seizure

Malar rash (*P* < 0.001), oral ulcer (*P* = 0.001), serositis (*P* < 0.001), renal involvement (*P* = 0.009), fever (*P* < 0.001), leukopenia (*P* < 0.001), and thrombocytopenia (*P* < 0.001) occurred significantly more frequently in NPSLE patients with seizure than in non-NPSLE patients. The SLEDAI-2K score, after the removal of NP scores, of NPSLE patients with seizure was significantly higher than that of non-NPSLE patients (13.3 ± 7.0 vs. 8.6 ± 6.7, *P* < 0.001). The positive rate of anti-ribosomal P protein antibodies for seizure patients was significantly higher than that for non-NPSLE patients (*P* = 0.001). Although there was no significant difference, the positive rate of anti-*β*2GP1 antibodies for seizure patients was higher than that for non-NPSLE patients (*P* = 0.058) (Table 5). This study also analyzed the clinical characteristics of NPSLE patients with seizure and those without seizure. Serositis (57.7% vs. 35.0%, *P* = 0.002) and thrombocytopenia (45.1% vs. 30.9%, *P* = 0.048) occurred significantly more frequently in NPSLE patients with seizure than in those without seizure. The SLEDAI-2K score of NPSLE patients with seizure was significantly higher than that of NPSLE patients without seizure (25.3 ± 8.8 vs 17.4 ± 8.0, *P* < 0.001) (Table 5). Among 71 seizure patients, 35 patients had diffuse NPSLE. There was no difference between seizure patients with diffuse NPSLE and those without diffuse NPSLE in the positivity of anti-ribosomal P protein antibodies (40.0% vs 42.9%, *P* = 0.808). Seventy-one NPSLE patients with seizure were followed up; of them, nine died. Thirteen of the 62 surviving patients were still using antiepileptic drugs at the last follow-up examination, and three patients had recurrent seizure symptoms within 6 months after the last follow-up examination.

### 3.5. Analysis of Cranial MRI of NPSLE Patients

Among 194 patients, cranial MRI data of 82 patients were finally collected and reviewed in detail by two experienced neuroradiologists. Among 82 patients, 50 (61.0%) had abnormal MRI results, including 44 (88.0%) SVD, 3 (6.0%) LVD, and 3 (6.0%) inflammatory disease. Thirty-nine (78.0%) patients had white matter hyperintensities (WMH); these were mainly focal and most often involved the frontal lobe. Three patients (6.0%) had lumen infarction, 7 (14.0%) had subcutaneous infarction, 6 (12.0%) had microhemorrhage on MRI, and 19 (38.0%) had brain atrophy. At the univariate analysis, patients with inflammatory lesions (*P* = 0.019) and LVD (*P* = 0.034) had shorter survival periods (Table 6). At multivariate analysis using Cox regression analysis, inflammatory lesions (*P* = 0.021), LVD (*P* = 0.038), proteinuria (*P* = 0.033), and elevated serum creatinine (*P* < 0.001) were independent prognostic factors of death.

## 4. Discussion

In this study, we analyzed the risk factors for the development of NP manifestations and prognosis factors for NPSLE in Chinese SLE patients. Higher disease activity and positive anti-ribosomal P antibody levels increased the risk of NPSLE development. NPSLE patients with renal insufficiency and higher SLEDAI scores had worse prognoses. We also analyzed the cranial MRI results of many patients. Patients with inflammatory lesions and LVD on MRI might have poor prognoses.

Nervous system involvement is common in SLE and is a major cause of morbidity and mortality [17]. The majority of patients with NPSLE were female in this study and in previous studies; this is because SLE more commonly occurs in women. The mean age of our patients at the onset of NPSLE was similar to the median age of 27.5 to 28 years reported in other studies [18]. In this study, NP syndromes usually occurred within 3 years after onset of SLE, which is consistent with other studies [19]. Some studies showed that NP syndromes can occur during the early stage of SLE [20, 21]. NPSLE commonly occurs in young and middle-aged patients, and it often occurs during the early stage of SLE. Therefore, treatment is urgently necessary during these times.

This study showed that NPSLE patients were more likely to also have other typical lupus symptoms, and that a combination of multiple symptoms indicates high disease activity [22]. Furthermore, our results showed that the SLEDAI-2K score of NPSLE patients is significantly higher than that of non-NPSLE patients, even after removing the scores related to NP symptoms. Patients are prone to NP symptoms during high lupus activity, and this result was consistent with that of previous studies [23, 24]. It has been reported that the presence of active disease and the presence of circulating autoantibodies are major risk factors for NP events [25]. In this study, leukopenia and thrombocytopenia were more likely to occur in NPSLE patients. Early observation suggested that thrombocytopenia was significantly and strikingly correlated with NP involvement in SLE patients [22]. Furthermore, it is widely accepted that antiribosomal P is related to NP involvement [26], which was confirmed by our results. Anti-RibP is more specifically associated with specific NPSLE manifestations, CNS involvement, depression, and psychosis [27]. Other antibodies, such as autoantibodies to NMDA receptor subunit NR2 (anti-NR2) [28] and anti-glucose-regulated protein 78 (anti-GRP78) [29], have been associated with the development of diffuse NPSLE. Also, the presence of anti-NMDA in cerebrospinal fluid but not in serum is associated significantly with overwhelming CNS abnormalities, suggesting the importance of direct access of autoantibodies to brain dysfunction [30]. However, it remains controversial whether the antibodies discussed above are important biomarkers for NPSLE; it may be advantageous to have more sensitive antigens to allow for earlier detection and treatment.

Previous studies reported that SLE patients are at higher risk for seizures than the general population [21, 31]. In this study, seizure was the most common subtype of NPSLE, which is consistent with the results of a SLICC study [32]. The pathogenesis of seizure in SLE is not clear; systemic inflammation, focal microvascular brain injuries, direct autoantibody effects on neuronal networks, and/or APS may all have a role [33, 34]. Other studies indicated that risk factors for seizures in SLE include disease activity, female sex, race, aPL level, and younger age [33, 35–37]. This study also confirmed that higher disease activity of lupus, positive anti-*β*2GP1, and younger age were risk factors for seizure in lupus.

It has been reported that NPSLE is a poor prognostic factor for SLE patients [21, 38, 39], which is consistent with our results. It has also been reported that ACS, seizure, and CVD in NPSLE are risk factors that affect survival [39]. Our results showed that NPSLE patients with high disease activity had decreased survival rates. Strong immunosuppressive treatments were used for NPSLE patients. However, aggressive treatment, including high doses of corticosteroids and strong immunosuppressants such as cyclophosphamide and mycophenolate mofetil may increase the risk of infection. Previous studies confirmed that the risk of opportunistic infection is higher in systemic lupus erythematosus patients treated with steroids than those not treated with steroids. Medium and high doses were associated with a higher risk of opportunistic infection compared with low doses [40, 41]. The most common cause of death in this study was infection, which is consistent with a previous study [19]. Therefore, research efforts are aggressively pursuing better treatment that can effectively control NPSLE and poses a relatively small risk of infection. Several cytokines have been preliminarily proven to be related to NPSLE. Agents for these specific cytokines are currently being explored, including type I IFN receptor inhibition [42], macrophage colony-stimulating factor 1 receptor (CSF1R) [43], and Bruton tyrosine kinase (BTK) inhibitor [44]. Furthermore, our result showed renal involvement, especially elevated Scr had decreased the survival rate of NPSLE patients, which was consistent with a previous study [19]. It was reported that lupus nephritis is a major risk factor for overall morbidity and mortality in SLE [45, 46], which could explain why elevated Scr is the risk factor of mortality in NPSLE.

Similar to a previous study, more than 30% of NPSLE patients had normal cranial MRI results [47]. Nearly half of the abnormal MRI results showed WMH, mainly involving the frontal lobe and parietooccipital lobe, which was also similar to other study results [48]. Inflammatory lesions occurred in 5.9% of patients who underwent MRI in this study. Other studies showed that the prevalence of inflammatory lesions ranged from 0% to 38.1% [23, 24]. A previous study showed that inflammatory lesions may be related to cerebral vasculitis in SLE patients, which can be diffuse or have focal distribution, and the large and small arteries can be involved [49]. A correlation between inflammatory lesions and clinical features was not found in this study, but the survival rate of patients with inflammatory lesions on MRI was significantly reduced. No similar study has focused on the relationship between inflammatory lesions and the prognosis for NPSLE. Another study has shown that abnormal cranial MRI results are poor prognostic factors for patients with diffuse NPSLE [50]; however, such results were not found in our study. Because a small number of patients underwent detailed MRI in this study, the results need to be verified by larger-scale research.

There were several limitations to this study. This was a retrospective study and the control group was relatively small, which could have affected the outcome and conclusions. Using hospitalized NPSLE patients may have led to a higher proportion of patients with severe cases. This study also had a short follow-up period. Longer-term studies are needed to investigate late mortality and organ damage of NPSLE patients. Furthermore, this was a single-center study; therefore, there was minimal ethnic variation.

## 5. Conclusions

High disease activity and positive rate of anti-ribosomal P protein antibodies may be risk factors for NPSLE. NPSLE decreases survival rates of SLE patients. Renal insufficiency and high disease activity are predictive of poor prognoses for NPSLE patients.

## Figures and Tables

**Table 1:** Baseline characteristics of the 194 patients with NPSLE.

Characteristics	Value (*n* = 194) (%)
Female	180 (92.8)
Age (years)	29.8 ± 10.8
SLE duration (years), mean ± SD	3.09 ± 4.40
NP duration (years), mean ± SD	2.78 ± 4.16
>1 NP subtype	71 (36.6)
Subtypes of NPSLE	
Central nervous system involvement	184 (94.8)
Seizure	71 (36.6)
Acute confusional state	49 (25.3)
Cerebrovascular disease	30 (15.5)
Headache	27 (13.9)
Psychosis	22 (11.3)
Cognitive impairment	19 (9.8)
Mood disorder	17 (8.8)
Demyelination	6 (3.1)
Dyskinesia	1 (0.5)
Myelitis	5 (2.6)
Aseptic meningitis	4 (2.1)
Anxiety disorder	3 (1.5)
Peripheral nervous system involvement	21 (10.8)
Cranial neuropathy	8 (15.5)
AIDP	3 (1.5)
Single/multiplex mononeuropathy	5 (3.6)
Polyneuropathy	5 (4.1)
Increased CSF pressure	73 (37.6)
Increased CSF protein	78 (40.2)
Treatment	
Steroid pulse	146 (75.3)
CTX	179 (92.3)
MMF	9 (4.6)

NPSLE: neuropsychiatric systemic lupus erythematosus; AIDP: acute inflammatory demyelinating polyradiculoneuropathy; CSF: cerebrospinal fluid; CTX: cyclophosphamide; MMF: mycophenolate mofetil.

**Table 2:** Comparison of clinical characteristics of the NPSLE and non-NPSLE groups.

	NPSLE group (*n* = 194) (%)	Non-NPSLE group (*n* = 220) (%)	*P* value
Female	180 (92.8)	202 (91.8)	0.714
Age (years), mean ± SD	29.86 ± 10.78	31.78 ± 11.25	0.397
SLE duration (years), mean ± SD	3.09 ± 4.40	2.81 ± 4.57	0.535
Malar rash	136 (70.1)	79 (35.9)	**<0.001**
Oral ulcer	39 (20.1)	17 (7.7)	**<0.001**
Alopecia	75 (38.7)	52 (23.6)	**0.001**
Arthritis	60 (30.9)	41 (18.6)	**0.004**
Vasculitis	12 (6.2)	20 (9.1)	0.269
Serositis	84 (43.3)	21 (9.5)	**<0.001**
Renal involvement	103 (53.1)	82 (37.3)	**0.001**
Fever	79 (40.7)	35 (15.9)	**<0.001**
Leukopenia	97 (50.0)	40 (18.2)	**<0.001**
Thrombocytopenia	84 (43.3)	31 (14.1)	**<0.001**
ESR (mm/h)	45.73 ± 32.79	33.29 ± 28.84	**<0.001**
CRP (mg/L)	16.72 ± 31.57	6.56 ± 10.02	**<0.001**
Hypocomplementemia	153 (78.9)	138 (62.7)	**<0.001**
Anti-dsDNA positive	97 (50.0)	116 (52.7)	0.580
SLEDAI	20.3 ± 9.1	8.6 ± 6.7	**<0.001**
SLEDAI-removed NP scores	12.3 ± 6.6	8.6 ± 6.7	**<0.001**
Immunologic disorder at enrollment			
Anti-Sm positive	69 (35.6)	114 (51.8)	**0.001**
Anti-RNP positive	101 (52.1)	121 (55.0)	0.489
Anti-SSA positive	126 (64.9)	151 (68.6)	0.436
Anti-SSB positive	34 (17.5)	72 (32.7)	**0.001**
Anti-ribosomal P protein positive	81 (41.8)	47 (21.4)	**<0.001**
Anticardiolipin positive	31 (16.6)	19 (11.7)	0.189
Lupus anticoagulant positive	38 (22.2)	44 (24.9)	0.562
Anti-*β*2 glycoprotein 1 positive	26 (14.3)	18 (9.6)	0.167

NPSLE: neuropsychiatric systemic lupus erythematosus; ESR: erythrocyte sedimentation rate; CRP: C-reactive protein; dsDNA: double-stranded deoxyribonucleic acid; SLEDAI: systemic lupus erythematosus disease activity index; NP: neuropsychiatric; RNP: ribonucleoprotein; Sm: Smith; SSA: Sjogren's syndrome antigen A; SSB: Sjogren's syndrome antigen B. Significant *P* values are shown in bold typeface.

**Table 3:** Prognostic analysis of clinical characteristics of NPSLE patients.

Risk factors	Dead group (*N* = 16), *n* (%)	Survival group (*N* = 178), *n* (%)	*χ* ^2^	*P* value
Female	15 (93.8)	165 (92.7)	0.049	0.824
Age older than 25 years	12 (75.0)	111 (62.4)	1.394	0.238
Seizure	9 (56.3)	62 (34.8)	2.945	0.086
Acute confusional state	5 (31.3)	44 (24.7)	0.345	0.557
Cerebrovascular disease	4 (25.0)	27 (15.2)	0.823	0.364
Psychosis	0 (0)	22 (12.4)	2.229	0.135
Rash	11 (68.8)	15 (70.2)	0.002	0.964
Oral ulcer	3 (18.8)	36 (20.2)	0.002	0.963
Vasculitis	0 (0)	12 (6.7)	1.099	0.294
Arthritis	8 (50.0)	52 (29.2)	2.795	0.095
Serositis	9 (56.3)	75 (42.1)	0.880	0.348
Respiratory involvement	5 (31.3)	28 (15.7)	2.422	0.120
Cardiac involvement	6 (37.5)	68 (38.2)	0.001	0.970
Renal involvement	12 (75.0)	90 (50.6)	3.108	0.078
Proteinuria	13 (81.3)	94 (52.8)	4.436	**0.035**
Elevated serum creatinine	6 (37.5)	19 (10.7)	10.584	**0.001**
Hematological involvement	10 (62.5)	103 (57.9)	0.156	0.693
Hypocomplementemia	16 (100)	137 (80.0)	4.666	**0.031**
Anti-dsDNA positive	9 (56.3)	88 (49.4)	0.273	0.602
Anti-Sm positive	6 (37.5)	64 (36.0)	0.031	0.861
Anti-RNP positive	8 (16.0)	93 (52.2)	0.045	0.831
Anti-SSA positive	10 (62.5)	116 (65.2)	0.004	0.953
Anti-SSB positive	2 (12.5)	32 (18.0)	0.296	0.587
Anti-ribosomal P protein positive	9 (56.3)	72 (40.4)	1.397	0.237
Anticardiolipin positive	1 (6.3)	30 (16.9)	1.042	0.307
Anti-*β*2 glycoprotein 1 positive	2 (12.5)	24 (13.5)	0.035	0.852
Lupus anticoagulant positive	1 (6.3)	37 (20.8)	1.616	0.204
SLEDAI ≥ 15	16 (100)	122 (68.5)	6.076	**0.014**
Elevated ESR	12 (75.0)	126 (70.8)	0.097	0.755
Elevated CRP	10 (93.8)	109 (61.2)	0.029	0.866
Increased CSF pressure	5 (31.3)	68 (38.2)	0.228	0.633
Increased CSF protein	5 (31.3)	73 (41.0)	0.358	0.550
Steroid pulse	13 (81.3)	133 (74.7)	0.363	0.547

NPSLE: neuropsychiatric systemic lupus erythematosus; dsDNA: double-stranded deoxyribonucleic acid; RNP: ribonucleoprotein; Sm: Smith; SSA: Sjogren's syndrome antigen A; SSB: Sjogren's syndrome antigen B; SLEDAI: systemic lupus erythematosus disease activity index; ESR: erythrocyte sedimentation rate; CRP: C-reactive protein; CSF: cerebrospinal fluid. Significant *P* values are shown in bold typeface.

**Table 4:** Cox's regression modeling of predictors of mortality.

Risk factor	HR (95% CI)	*P* value
Proteinuria	2.16 (0.54-8.59)	0.276
Elevated serum creatinine	3.27 (1.13-9.45)	**0.029**
SLEDAI ≥ 15	1.71 (0.57 -5.14)	0.340

HR: hazard ratio; CI: confidence interval; SLEDAI: systemic lupus erythematosus disease activity index. A multivariate Cox proportional hazard model was employed to identify independent predictors of survival. Significant *P* values are shown in bold typeface.

**Table 5:** Comparison of clinical characteristics of NPSLE patients with seizure, NPSLE patients without seizure, and non-NPSLE patients.

	NPSLE with seizure, (*n* = 71) (%)	Non-NPSLE (*n* = 220) (%)	*P* value	NPSLE without seizure, (*n* = 123) (%)	*P* value
Female	66 (93.0)	202 (91.8)	0.757	114 (92.7)	0.943
Age (years)	26.20 ± 8.41	30.78 ± 11.25	**0.002**	32.0 ± 11.4	**<0.001**
SLE duration (years)	2.66 ± 3.71	2.81 ± 4.57	0.796	3.3 ± 4.7	0.304
Malar rash	51 (71.8)	79 (35.9)	**<0.001**	85 (69.1)	0.690
Oral ulcer	16 (22.5)	17 (7.7)	**0.001**	23 (18.7)	0.521
Arthritis	21 (29.6)	41 (18.6)	0.050	39 (31.7)	0.757
Serositis	41 (57.7)	21 (9.5)	**<0.001**	43 (35.0)	**0.002**
Vasculitis	5 (7.0)	20 (9.1)	0.592	7 (5.7)	0.707
Renal involvement	39 (54.9)	82 (37.3)	**0.009**	64 (52.0)	0.697
Fever	30 (42.3)	35 (15.9)	**<0.001**	49 (39.8)	0.741
Leukopenia	37 (52.1)	46 (20.9)	**<0.001**	48 (39.0)	0.077
Thrombocytopenia	32 (45.1)	33 (15.0)	**<0.001**	38 (30.9)	**0.048**
ESR (mm/h)	49.15 ± 33.20	33.29 ± 28.84	**<0.001**	43.72 ± 32.52	0.296
CRP (mg/L)	18.55 ± 36.83	6.56 ± 10.02	**<0.001**	15.65 ± 28.17	0.540
Hypocomplementemia	57 (80.3)	138 (62.7)	**0.006**	96 (78.0)	0.714
Anti-dsDNA positive	33 (46.5)	116 (52.7)	0.360	58 (47.2)	0.506
Anti-Sm positive	27 (38.6)	114 (51.8)	**0.043**	43 (35.0)	0.616
Anti-RNP positive	38 (54.3)	121 (55.0)	0.210	63 (51.2)	0.628
Anti-SSA positive	42 (60.0)	151 (68.6)	0.087	84 (68.3)	0.245
Anti-SSB positive	11 (15.7)	72 (32.7)	**0.005**	23 (18.7)	0.601
Anti-RibP positive	29 (40.8)	47 (21.4)	**0.001**	52 (42.6)	0.872
aCL positive	11/67 (16.4)	19/163 (11.7)	0.330	20/120 (16.7)	0.965
LA positive	13/62 (21.0)	44 (24.9)	0.536	25/109 (22.9)	0.766
Anti-*β*2GP1 positive	12/65 (18.5)	18/187 (9.6)	0.058	14/117 (12.0)	0.230
SLEDAI	25.3 ± 8.8	8.6 ± 6.7	**<0.001**	17.4 ± 8.0	**<0.001**
SLEDAI without NP scores	13.3 ± 7.0	8.6 ± 6.7	**<0.001**	11.7 ± 6.6	0.105

NPSLE: neuropsychiatric systemic lupus erythematosus; ESR: erythrocyte sedimentation rate; CRP: C-reactive protein; dsDNA: double-stranded deoxyribonucleic acid; RNP: ribonucleoprotein; Sm: Smith; SSA: Sjogren's syndrome antigen A; SSB: Sjogren's syndrome antigen B; RibP: ribosomal P protein; aCL: anticardiolipin; LA: lupus anticoagulant; *β*2GP1: *β*2 glycoprotein 1; SLEDAI: systemic lupus erythematosus disease activity index. Significant *P* values are shown in bold typeface.

**Table 6:** Prognostic analysis of neuroimaging results of NPSLE patients.

Risk factor	Dead group (*n* = 5), *n* (%)	Survival group (*n* = 77), *n* (%)	*χ* ^2^	*P*
Abnormal MRI	3 (60.0%)	47 (61.0%)	0.006	0.936
Inflammatory lesions	1 (20.0%)	2 (2.6%)	5.516	**0.019**
Large vessel disease	1 (20.0%)	2 (2.6%)	4.512	**0.034**
Small vessel disease	1 (20.0%)	43 (55.8%)	2.453	0.117
White matter hyperintensities	2 (40.0%)	37 (48.1%)	0.129	0.720
Lacunes	0 (0)	3 (3.9%)	0.196	0.658
Subcutaneous infarction	1 (20.0%)	6 (7.8%)	0.919	0.338
Microbleeds	0 (0)	6 (7.8%)	0.408	0.523
Brain atrophy	1 (20.0%)	18 (23.4%)	0.040	0.841

NPSLE: neuropsychiatric systemic lupus erythematosus; MRI: magnetic resonance imaging. Significant *P* values are shown in bold typeface.

## Data Availability

The data used to support the findings of this study are available from the corresponding authors upon request.
